# Dysfunctions Associated with Methylation, MicroRNA Expression and Gene Expression in Lung Cancer

**DOI:** 10.1371/journal.pone.0043441

**Published:** 2012-08-17

**Authors:** Tao Huang, Min Jiang, Xiangyin Kong, Yu-Dong Cai

**Affiliations:** 1 Institute of Systems Biology, Shanghai University, Shanghai, People's Republic of China; 2 Key Laboratory of Systems Biology, Shanghai Institutes for Biological Sciences, Chinese Academy of Sciences, Shanghai, People's Republic of China; 3 Shanghai Center for Bioinformation Technology, Shanghai, People's Republic of China; 4 Institute of Health Sciences, Shanghai Institutes for Biological Sciences, Chinese Academy of Sciences and Shanghai Jiao Tong University School of Medicine, Shanghai, People's Republic of China; 5 State Key Laboratory of Medical Genomics, Ruijin Hospital, Shanghai Jiaotong University, Shanghai, People's Republic of China; Southern Illinois University School of Medicine, United States of America

## Abstract

Integrating high-throughput data obtained from different molecular levels is essential for understanding the mechanisms of complex diseases such as cancer. In this study, we integrated the methylation, microRNA and mRNA data from lung cancer tissues and normal lung tissues using functional gene sets. For each Gene Ontology (GO) term, three sets were defined: the methylation set, the microRNA set and the mRNA set. The discriminating ability of each gene set was represented by the Matthews correlation coefficient (MCC), as evaluated by leave-one-out cross-validation (LOOCV). Next, the MCCs in the methylation sets, the microRNA sets and the mRNA sets were ranked. By comparing the MCC ranks of methylation, microRNA and mRNA for each GO term, we classified the GO sets into six groups and identified the dysfunctional methylation, microRNA and mRNA gene sets in lung cancer. Our results provide a systematic view of the functional alterations during tumorigenesis that may help to elucidate the mechanisms of lung cancer and lead to improved treatments for patients.

## Introduction

Cancer is a systems biology disease [1] that involves the dysregulation of multiple pathways at multiple levels [2]. High-throughput technologies, such as genomic sequencing and transcriptomic, proteomic and metabolomic profiling, have provided large quantities of experimental data. However, systems biology requires not only new high-throughput “-omics” data-generation technologies but also integrative analysis methods that may shed light on the potential mechanisms of complex diseases. Lung cancer is one of the leading causes of cancer death worldwide [3]. There are currently known genetic, epigenetic, transcriptomic, proteomic, metabolomic, and microRNA markers of lung cancer [4]. Because epigenetic changes occur early during tumorigenesis, methylation markers should be considered [4]. The protein is the final, functional form of the genetic information; therefore, proteomic markers are also important. Transcriptomic markers are easy to measure, and mRNA levels are frequently used as a proxy for protein abundance [5]. MicroRNA, as an important regulatory contributor, is also an excellent lung cancer biomarker [6], [7]. Whether a methylation marker, mRNA marker, or microRNA marker is considered, these markers function by affecting biological pathways or networks. The functional pathways are the common bridges between various markers and the disease.

Currently, there are several studies on multi-dimensional data integration [8]–[11]. Most of them were based on regression between different dimensions [10] and require each sample to have multiple level data [11]. The dysfunctional pathways were identified by enrichment analysis of aberrant genes [9].

In this study, we directly analyze dysfunctions of non-small-cell lung cancer (NSCLC) by comparing the functional sets of methylation, microRNA and mRNA data between lung cancer tissues and normal lung tissues. Each functional set corresponds to one Gene Ontology (GO) [12] term. Three sets of this functional unit are defined: the methylation set, the microRNA set and the mRNA set. The Matthews correlation coefficient (MCC), evaluated by leave-one-out cross-validation (LOOCV), is used to represent the discriminating ability of each gene set. The MCC ranks of each methylation set, microRNA set and mRNA set are analyzed. Six groups of GO sets are classified, and 20 dysfunctional methylation, microRNA and mRNA gene sets in lung cancer are identified. These dysfunctional sets characterize the processes of tumorigenesis. With an accurate characterization of tumorigenesis, we may better understand the mechanisms of lung cancer and improve the early diagnosis, treatment efficiency evaluation, and prognosis of lung cancer.

## Materials and Methods

### Data sets

We downloaded the methylation profiles of 1,413 genes in 57 NSCLC patients and 52 control samples [13] from GEO (Gene Expression Omnibus) with the accession number GSE16559. The microRNA expression profiles of 549 microRNAs in 187 NSCLC patients and 188 control samples [14] were retrieved from GEO with the accession number GSE15008. The mRNA gene expression profiles of 19,700 genes in 46 NSCLC patients and 45 control samples [15] were obtained from GEO with the accession number GSE18842.

Since the methylation data, microRNA data and mRNA data were obtained from different NSCLC studies, we compared the clinical information of patients from these three studies. The two kinds of clinical information that were given in at least two studies were age and grade of differentiation. The clinical information from these three studies is shown in **Table 1**. The average age of patients from the methylation study is 68.2 and their standard deviation is 11.4; meanwhile, the average age of patients from the microRNA study is 59.9 and the standard deviation is 9.8. The ages of patients in these two studies are similar. The percentages of well-, moderately- and poorly-differentiated cancer patients in the microRNA study and the mRNA study were
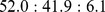
and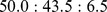, respectively. The distributions of grades of differentiation in these two studies were very similar. Based on the available clinical information on these NSCLC patients, we think that these three data sets may represent some common dysfunctions of NSCLC.

**Table 1:** Clinical information for NSCLC patients in three data sets.

	Methylation data	microRNA data	mRNA data
Age: Mean (Standard Deviation)	68.2 (11.4)s	59.9 (9.8)	-
Differentiation: Well, %	-	52.0	50.0
Differentiation: Moderate, %	-	41.9	43.5
Differentiation: Poor, %	-	6.1	6.5

### The target genes of microRNAs

We define the target genes of the microRNAs to be those that were predicted by at least three out of the following six software tools: miRBase [16] (http://microrna.sanger.ac.uk/targets/v5/), TargetScan [17] (http://www.targetscan.org/), miRanda [18] (http://www.microrna.org/microrna/), TarBase [19] (http://diana.cslab.ece.ntua.gr/tarbase/), mirTarget2 [20] (http://mirdb.org/miRDB/download.html), and PicTar [21] (http://pictar.mdc-berlin.de/). **Table S1** gives the microRNA - target gene pairs that are predicted by at least three tools.

### The GO gene sets for methylation, microRNA and mRNA

For each GO term, we define three gene sets to represent it: first, the methylation gene set, which consists of the genes that are annotated to the GO term and for which the methylation level has been measured; second, the microRNA gene set, which consists of the microRNAs that have target genes annotated to this term; and third, the mRNA gene set, which consists of all the genes annotated to this term.

### The discriminating ability of gene sets

We evaluated the discriminating ability of gene sets by constructing a prediction model. First, the Nearest Neighbor Algorithm (NNA) [5], [22]–[30] was applied to build the prediction model. Next, the prediction models were tested using LOOCV [5], [22]–[31]. Finally, the Matthews correlation coefficient (MCC) [26], [30] of LOOCV was used as the measurement of the gene set's discriminating ability.

The NNA [5], [22]–[30] is a widely used machine learning method. The NNA makes its prediction by comparing the distances between the query sample and the samples with known classes, i.e., the lung cancer samples or control samples. The query sample was predicted to have the same class as its nearest neighbor, i.e., the sample with known class that has the smallest distance. In this analysis, the distance between two samples 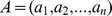 and 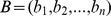 was defined as one minus the cosine similarity between the two samples [5], [23]–[27], [30], [32]–[34]:
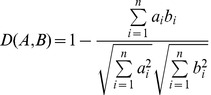
(1)


The NNA program can be downloaded from http://pcal.biosino.org/NNA.html.

During LOOCV [32], [35], [36], each sample in the benchmark dataset will be chosen as the test set once and tested by the prediction model trained by the rest of the samples.

The Matthews correlation coefficient (MCC) is a balanced measurement of prediction performance that considers both sensitivity and specificity [26], [30]. It is calculated using the following formula:
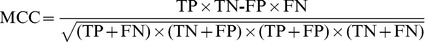
(2)in which TP, TN, FP and FN are the numbers of true lung cancer samples, true control samples, false lung cancer samples and false control samples, respectively.

### Classification of gene sets based on their dysfunctional level: methylation, microRNA or mRNA

After we calculated the MCC of each gene set at each level, we ranked the gene sets of each level based on their MCCs and compared the ranks of the three levels, methylation, microRNA and mRNA, in each gene set. With certain values proving to be equal, their ranks were replaced by their mean ranks. As an example of a GO term, if its methylation level had changed between normal and cancer tissue, but its microRNA and mRNA levels had not changed, it was defined as a methylation dysfunctional GO gene set. Similarly, we can define other types of GO gene sets. In total, we defined six groups of gene sets, one for each possible rank ordering of methylation, microRNA and mRNA.

### The work flow of dysfunctional methylation, microRNA and mRNA gene set analysis

**Figure 1:**
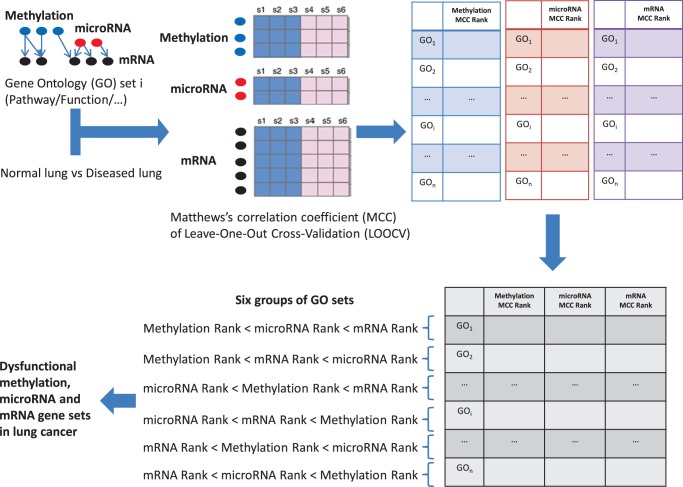
The work flow of dysfunctional methylation, microRNA and mRNA gene set analysis. First, for each Gene Ontology (GO) term, we defined three gene sets: the methylation set, the microRNA set and the mRNA set. Next, we calculated the Matthews's correlation coefficient (MCC), as evaluated by leave-one-out cross-validation (LOOCV), for each gene set. Next, we ranked the MCCs in the methylation sets, the microRNA sets and the mRNA sets, and we compared the MCC ranks of methylation, microRNA and mRNA for each Gene Ontology (GO) term and classified the GO sets into six groups. Finally, we identified the dysfunctional methylation, microRNA and mRNA gene sets in lung cancer.

Our strategy of dysfunctional methylation, microRNA and mRNA gene set analysis is demonstrated in **Figure** **1**. First, for each GO term, we defined three sets: the methylation set, the microRNA set and the mRNA set. Next, we calculated each gene set's MCC, as evaluated by LOOCV. We ranked the MCCs in the methylation sets, the microRNA sets and the mRNA sets. Next, we compared the MCC ranks of methylation, microRNA and mRNA in each GO term and classified the GO sets into six groups based on these ranks. Finally, we identified the dysfunctional methylation, microRNA and mRNA gene sets in lung cancer.

## Results and Discussion

### The GO gene sets of methylation, microRNA and mRNA

We cross-referenced the three data sets that measured the methylation, microRNA and mRNA of lung cancer tissues and control tissues with GO and found 4,381 GO gene sets that have methylation, microRNA and mRNA data. The three levels of gene sets for these 4,381 GO terms were compiled as follows: the methylation set for each GO term consists of the genes that had methylation data and were annotated to this term, the microRNA set consists of the microRNAs that had target genes annotated to this term, and the mRNA set consists of all of the genes that were annotated to this term. The 4,381 GO sets of mRNA, microRNA and methylation can be found in **Dataset S1**, **Dataset S2** and **Dataset S3**, respectively.

### The discriminating ability of the methylation, microRNA and mRNA gene sets

**Figure 2:**
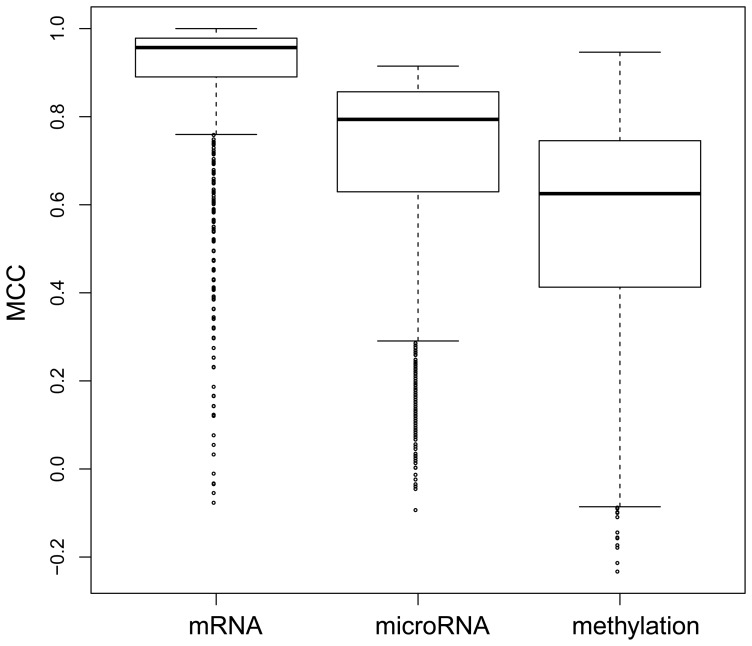
The MCC boxplot of methylation, microRNA and mRNA gene sets. The mean MCCs of the mRNA, microRNA and methylation gene sets were 0.897, 0.702 and 0.561, respectively. The MCCs of the mRNA sets were significantly greater than the MCCs of the microRNA sets with a one-sided t-test p-value of less than 2.2e-16, and the MCCs of the microRNA sets were, in turn, significantly greater than the MCCs of the methylation sets with a one-sided t-test p-value of less than 2.2e-16.

We measured the ability of the gene sets to discriminate between cancer and normal tissue using the Matthews correlation coefficient (MCC) of the NNA prediction model evaluated by LOOCV. We compared the MCCs of methylation, microRNA and mRNA. **Figure 2** shows the MCC distributions of the methylation, microRNA and mRNA gene sets. The mean MCCs of the mRNA, microRNA and methylation gene sets are 0.897, 0.702 and 0.561, respectively. The one-side-greater t-test p-value for the mRNA and microRNA sets is less than 2.2e-16. The one-side-greater t-test p-value for the microRNA and methylation sets is also less than 2.2e-16. These results indicate that the MCCs of the mRNA sets are significantly greater than the MCCs of the microRNA sets, which are, in turn, significantly greater than the MCCs of the methylation sets.

**Figure 3:**
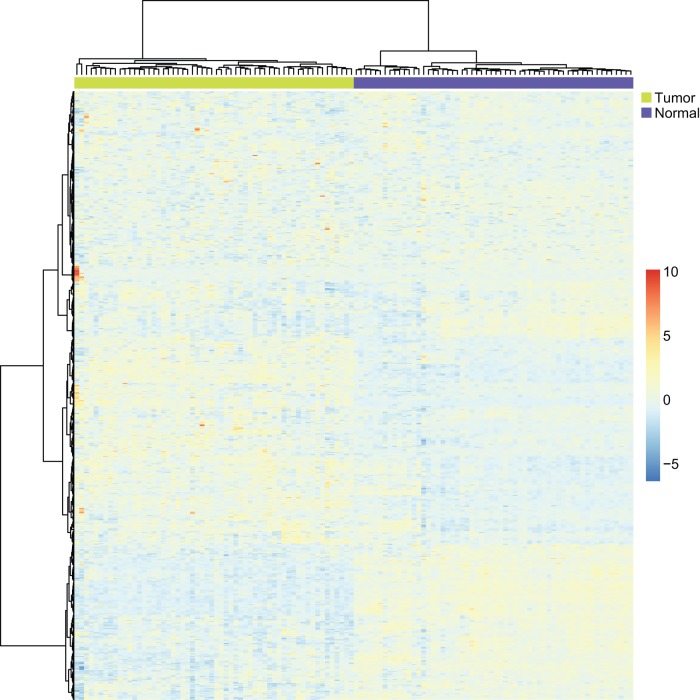
The heatmap of the high frequency genes and the tumor/normal samples. The green bars indicate the tumor samples and the blue bars indicate the normal samples. The tumor and normal samples were clearly differentiated by the high frequency genes.

**Figure 4:**
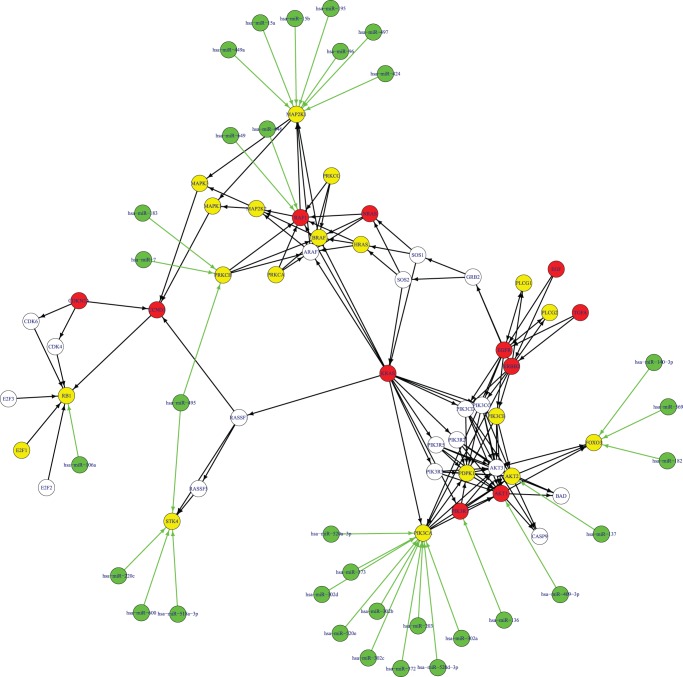
The high frequency genes and microRNAs of the KEGG pathway “hsa05223 Non-small cell lung cancer”. The green nodes denote high frequency microRNAs. The red nodes denote high frequency genes in both methylation and mRNA dysfunctional sets. The yellow nodes indicate high frequency genes in mRNA dysfunctional sets only. There is no specific high frequency gene in methylation dysfunctional sets. The white nodes indicate non-high frequency genes. The black edges show interactions from the KEGG pathway “hsa05223 Non-small cell lung cancer”. The green edges show regulation by high frequency microRNAs on their target genes.

### Classification of gene sets based on their dysfunctional level: methylation, microRNA or mRNA

By comparing the MCC ranks of the gene sets at the methylation, microRNA or mRNA level, we defined six groups of gene sets. There are 960 gene sets in which methylation rank < microRNA rank < mRNA rank; 638 gene sets in which methylation rank < mRNA rank < microRNA rank; 721 gene sets in which microRNA rank < methylation rank < mRNA rank; 684 gene sets in which microRNA rank < mRNA rank < methylation rank; 584 gene sets in which mRNA rank < methylation rank < microRNA rank; and 794 gene sets in which mRNA rank < microRNA rank < methylation rank. **Table S2** shows the methylation, microRNA and mRNA dysfunction groups of the 4,381 GO gene sets.

### The dysfunctional gene sets in lung cancer

We ranked the dysfunctional gene sets in lung cancer based on the summed MCC ranks of methylation, microRNA and mRNA. The top 20 dysfunctional gene sets in lung cancer shown in **Table S3** were analyzed. These 20 dysfunctional gene sets in lung cancer are GO:0048585 (negative regulation of response to stimulus), GO:0007517 (muscle organ development), GO:0048514 (blood vessel morphogenesis), GO:0051146 (striated muscle cell differentiation), GO:0001525 (angiogenesis), GO:0045595 (regulation of cell differentiation), GO:0007162 (negative regulation of cell adhesion), GO:0060191 (regulation of lipase activity), GO:0006275 (regulation of DNA replication), GO:0061061 (muscle structure development), GO:0022008 (neurogenesis), GO:0008543 (fibroblast growth factor receptor signaling pathway), GO:0035107 (appendage morphogenesis), GO:0035108 (limb morphogenesis), GO:0001568 (blood vessel development), GO:0005576 (extracellular region), GO:0050793( regulation of developmental processes), GO:0010648 (negative regulation of cell communication), GO:0023057 (negative regulation of signaling), and GO:0019216 (regulation of lipid metabolic processes). Many of these GO terms have been reported to be associated with lung cancer. We analyze several GO sets as examples.

#### GO:0045595 (regulation of cell differentiation, ranked 6^th^) and GO:0050793 (regulation of developmental processes, ranked 17^th^)

Developmental processes and cell differentiation are regulated by a series of similar genes in normal tissues. Therefore, changes in these genes are frequently associated with carcinogenesis. Naveen Babbar et al. reported that TNFα can activate NFκB signaling in NSCLC cells [37], which results in decreased cell growth and increased apoptosis [37]. A role for FGF/FGFR family members has also been indicated in lung cancer. For example, frequent amplification of FGFR1 was identified in human squamous cell lung cancer [38]. Additionally, somatic mutations in several of these genes were identified in lung carcinomas, including FGFR1, FGFR2, and FGF2/10 [39]–[42]. Usually, tumor suppressor genes, such as P53, CDKN2A/B, and STK11, are downregulated, and oncogenes (such as KRAS and ERBB2/4) are upregulated in lung cancer [40]. MicroRNAs are involved in lung cancer due to the epigenetic changes that occur in cancer cells. The low expression of miR-200 and miR-205 is associated with the epithelial-mesenchymal transition (EMT) and stem-cell-like properties of cancer cells and promotes invasion and translocation [43]–[45]. The enforced expression of miR-29 family members in lung cancer cells can restore normal patterns of DNA methylation, induce the re-expression of methylation-silenced tumor suppressor genes, such as FHIT and WWOX, and inhibit tumorigenicity [46].

#### GO:0022008 (neurogenesis, ranked 11^th^)

Several genes annotated to this GO term are associated with acantha and brain metastases; for example, mutations in activating epidermal growth factor receptor (EGFR) were found in many lung cancer patients [47]. Human lung cancer features extensive alterations of microRNA expression that may deregulate cancer-related genes; for example, hsa-miR-125a-5p silencing unregulated ROCK1, miR-34b methylation caused c-Met overexpression, and miR-200c was silenced by methylation and downregulated TCF8 and E-cadherin, which resulted in cancer invasion and deterioration [48]–[50]. Demethylation and mutation of genes (ERBB2, KRAS) can also cause carcinogenesis [51], [52]. Methylation of the Death-associated protein kinase (DAPK) promoter and the opioid binding protein/cell adhesion molecule-like gene (OPCML) has been found in both adenocarcinoma and squamous-cell carcinoma [53], [54].

#### GO:0005576 (extracellular region, ranked 16^th^)

Epithelial Mesenchymal Transition (EMT) is the main process required for tumor invasion and translocation. Mutations in TIMP3, LAMA/B/C, TMEFF2, CDH13 and other genes are involved in lung cancer deterioration [55]. IL-8 can initiate an airway epithelial signaling pathway, and deregulation of this gene may cause tobacco-related lung cancer [56]. Five microRNAs (hsa-miR-155, hsa-miR-17-3p, hsa-let-7a-2, hsa-miR-145, and hsa-miR-21) are seen to be expressed differently in lung cancer tissues versus the corresponding noncancerous lung tissues. Among these microRNAs, let-7a can regulate RAS activity [57]. Epigenetic activation of human kallikrein 13 (KLK13) enhances the malignancy of lung adenocarcinoma by promoting N-cadherin expression and laminin degradation [58]. Recently, MMP1 was reported to be associated with lung cancer. The -16071G-2G polymorphism of MMP1 results in transcriptional up regulation [58]. X Xiang et al. reported that the stable expression of miR-155 significantly reduces the aggressiveness of tumor cell dissemination by preventing the EMT of tumor cells in vivo [59]. Furthermore, miR-155 directly suppresses the expression of the transcription factor TCF4, which is an important regulator of EMT [59].

### The high frequency genes and microRNAs in the top dysfunctional gene sets

We calculated the frequency of genes or microRNAs in the top 300 dysfunctional gene sets. The genes in either mRNA or methylation gene sets with frequency higher than 50 were defined as high frequency genes. Similarly, the high frequency microRNAs were defined as microRNAs that have frequency higher than 50 in the top 300 dysfunctional gene sets. The high frequency genes and microRNAs are given in **Table S4**.

We tested the discriminating ability of these high frequency genes in an independent data set which includes 58 lung cancer samples and 58 adjacent normal samples. The independent data set was downloaded from GEO with the accession number GSE32863. It was found that the high frequency genes can perfectly differentiate the lung cancer tissues from adjacent normal tissues. The prediction MCC was 1, which means that all samples were correctly classified in their actual group, tumor or normal. The heatmap of the high frequency genes and the tumor/normal samples is shown in **Figure 3**. The tumor and normal samples were clearly differentiated by the high frequency genes.

We did a hypergeometric test [5], [24], [25], [32], [36] to investigate whether the high frequency genes are significantly overlapped with the KEGG pathway “hsa05223 Non-small cell lung cancer”. The hypergeometric test p value was a highly significant 1.61E-26. This result suggests that many higher frequency genes are known “hsa05223 Non-small cell lung cancer” genes.

In **Figure 4**, we highlighted the high frequency genes we discovered in the KEGG pathway “hsa05223 Non-small cell lung cancer”. Many hub genes of the KEGG pathway “hsa05223 Non-small cell lung cancer” were high frequency dysfunctional genes, such as KRAS, EGFR, ERBB2, CDKN2A and RB1. And the hub high frequency genes tend to be dysfunctional at both the methylation and mRNA levels. It is known that KRAS can initiate tumorgenesis by affecting the endodermal progenitor [60]. The copy number alterations of KRAS are strongly associated with clinical outcomes of lung cancer patients [61]. EGFR is a receptor of the epidermal growth factor family. Binding of EGFR to a ligand will induce cell proliferation [62]. EGFR mutations are very common in lung cancer [63] and are associated with prognosis of NSCLC [64]. They can alter the signaling cascades of NSCLC [65]. ERBB2 is mutated in 4% of NSCLC [66] and its polymorphisms increase the risk of lung cancer [67]. Methylation of CDKN2A occurs more frequently in NSCLC tissues than in non-tumor tissues [68]. CDKN2A is involved in the p16/pRb/cyclin-D1 pathway [69]. RB1 can regulate cell proliferation, differentiation, and apoptosis in human NSCLC [70]. In advanced NSCLC patients, the frequency of Rb loss is high [71].

In **Figure 4**, there are some high frequency microRNAs, such as hsa-miR-495, hsa-miR-96, has-miR-106a, has-miR-137, has-miR-372, hsa-miR-183, hsa-miR-182, hsa-miR-203, hsa-miR-15a, hsa-miR-15b and hsa-miR-7. hsa-miR-495 regulates two high frequency dysfunctional genes, STK4 and PRKCB. It was reported that miR-495 is upregulated in KRAS-positive NSCLC [72]. hsa-miR-96 is downregulated in NSCLC [73]. has-miR-106a is related to lung cancer patient survival [56]. Patients with high expression of has-miR-106a tend to have a worse prognosis [56]. has-miR-137 and has-miR-372 are both upregulated in NSCLC and their expression levels are associated with survival and relapse in NSCLC patients [74]. has-miR-183 is a potential metastasis-inhibitor of lung cancer and can regulate migration and invasion genes [75]. hsa-miR-183 and hsa-miR-182 were reported as the most differentially expressed microRNAs between lung cancer tissues with adjacent normal tissues [76]. hsa-miR-203 is upregulated in lung cancer tissues [56]. hsa-miR-15a is frequently deleted or down-regulated in NSCLC [77] and its expression inversely correlates with the expression of cyclin D1[77]. hsa-miR-15 b is differentially expressed in tumor necrosis factor (TNF)-related apoptosis-inducing ligand (TRAIL) resistant NSCLC cells [73]. hsa-miR-7 is downregulated in lung cancer and it can regulate epidermal growth factor receptor signaling [78].

### The advantages and limitations of our methods

Obtaining a systematic understanding of pathological change is an essential problem in medical and pharmaceutical studies. Tumorigenesis involves alterations to many proteins, molecules and pathways. Eventually, however, all these changes cause cancer through functional effects. In this study, we used GO to describe biological functions and stratified the functions into three levels: methylation, microRNA and mRNA. In each level, we calculated and ranked the discriminating ability of the functional set for this level that was measured by the MCC correctly classifying cancer and normal tissues. For each functional set, we compared the MCC rank of each level, and we subsequently grouped the functional sets into six patterns based on the relationships of the MCC ranks of the different levels. Some functional sets may function at the methylation level; others may function at the microRNA level. Taking all three levels into consideration, we ranked the functional sets based on their overall ranks on the three levels. The overall ranking of the functional sets appears reasonable and is consistent with several published studies.

There are still several limitations to this research. Firstly, the methylation, microRNA and mRNA data for lung cancer and normal tissues are obtained from different studies, which may affect the results. Ideally, all of the data would be derived from the same study. To partially overcome this problem, we used the MCC rank, instead of the MCC itself, when comparing among the different levels. Secondly, the links between microRNAs and their target genes are based on predictions. Due to the low proportion of experimentally confirmed microRNA and target gene pairs, we used the microRNA and target gene pairs that were predicted by at least three popular microRNA target-gene predictors. Thirdly, not all functional sets were analyzed. The methylation, microRNA and mRNA data we used were generated with microarray technology. Certain genes or microRNAs were not measured, especially with respect to the methylation status of genes. With the development of sequencing technology and sequence capture technology, increasing numbers of genes can be measured, allowing us to analyze more functional sets and obtain a more comprehensive view of tumorigenesis.

Overall, our methods provide a means of performing “multi-omics” dysfunctional set analysis, which could be useful in the study of complex diseases. Our results yield a systematic view of tumorigenesis that may shed light on the diagnosis and prognosis of lung cancer.

## Supporting Information

Dataset S1 **The 4,381 Gene Ontology (GO) sets of mRNA.** Each line describes a gene set. The first field contains the Gene Ontology (GO) term name, the second field contains the number of mRNAs in the set, and the remaining fields list the mRNAs in the set.
(TXT)

Dataset S2 **The 4,381 Gene Ontology (GO) sets of microRNA.** Each line describes a microRNA set. The first field contains the Gene Ontology (GO) term name, the second field contains the number of microRNAs in the set, and the remaining fields list the microRNAs in the set.
(TXT)

Dataset S3 **The 4,381 Gene Ontology (GO) sets of methylation.** Each line describes a gene set. The first field contains the Gene Ontology (GO) term name, the second field contains the number of genes in the set, and the remaining fields list the genes in the set.
(TXT)

Table S1 **The microRNA - target gene pairs that were predicted by at least three tools.**
(XLSX)

Table S2 **The methylation, microRNA and mRNA dysfunction groups of the 4,381 Gene Ontology (GO) gene sets.**
(XLSX)

Table S3 **The top 20 dysfunctional gene sets in lung cancer.**
(PDF)

Table S4 **The high frequency genes and microRNAs.**
(XLSX)
