# Supplementary material for: Dysfunctions Associated with Methylation, MicroRNA Expression and Gene Expression in Lung Cancer
Source: PLoS One. 2012 Aug 17;7(8):e43441. doi: 10.1371/journal.pone.0043441 (PMC3422260; doi:10.1371/journal.pone.0043441)
Supplement: Table S3 — The top 20 dysfunctional gene sets in lung cancer. (PDF) [file pone.0043441.s006.pdf]

Table S3 - The top 20 dysfunctional gene sets in lung cancer.

Rank	GO term	Lung cancer associated mRNA	Lung cancer associated microRNA	Lung cancer associated methylation	Relationships among mRNA, microRNA and methylation in lung cancer
1	GO:0048585_negative regulation of response to stimulus	TNF-a[1], IL8[2], NFKBIA, NFKBID, SFRP1, WWOX, SLIT2[3]	hsa-miR-125a-3p, hsa-miR-155[4], hsa-let-7a, hsa-miR-200b/c, hsa-miR-205[5, 6], hsa-miR-29 a/b/c[7]	IL8, SFRP1, SLIT2	The enforced expression of miR-29s in lung cancer cell can restore normal patterns of DNA methylation, induces reexpression of methylation -silenced tumor suppressor genes, such as FHIT and WWOX, and inhibits tumorigenicity[7]
2	GO:0007517_muscle organ development	CHRNA1, ERBB2, TWIST1[8]	hsa-miR-125a-3p, hsa-miR-155[9], hsa-miR-200b/c, hsa-miR-205, hsa-miR-29a/b/c	ERBB2, TWIST1	
3	GO:0048514_blood vessel morphogenesis	ERBB2, CDH13, SFRP1, EGFL7, VEGFA, SLIT2	Hsa-miR-375, hsa-miR-34b, hsa-miR-155, hsa-miR-200b/c, hsa-miR-205, hsa-miR-29a/b/c	ERBB2, SFRP1, SLIT2	Promoters of miR-34b, EGFL7 are methylation, up regulate c-Met and Crk expressions[10]
4	GO:0051146_striated muscle cell differentiation	CHRNA1, ERBB2	hsa-miR-125a-3p, hsa-miR-155	ERBB2	
5	GO:0001525_angiogenesis	VEGFC[11], IL8, ERBB2, CDH13, SFRP1, EGFL7, VEGFA, SLIT2	Hsa-miR-375, hsa-miR-155, hsa-miR-200b/c, hsa-miR-205, hsa-miR-29a/b/c	IL8, ERBB2, CDH13, SFRP1, SLIT2	
6	GO:0045595_regulation of cell differentiation	VEGFC NFKB1, NFKBIA, NFKBID, TNF, KEAP1, CHRNA3, ERBB2, CDKN2A , FAS, NEUROD1, CDX2, SFRP1, TWIST1, DNMT3B, SLIT2	hsa-miR-125a-5p, hsa-miR-125a-3p, hsa-miR-34b, hsa-miR-155, hsa-miR-200b/c, hsa-miR-205, hsa-miR-29 a/b/c[12]	NFKB1[1], TNF, ERBB2, CDKN2A , FAS, NEUROD1, SFRP1, TWIST1, CALCA, DNMT3B, SLIT2[13, 14]	miR-200c was silenced by methylation and down regulated TCF8 and E-cadherin, which result in cancer invasion and deterioration[5]; up regulation of miR-29 make sure normal patterns of DNA methylation, induces reexpression of methylation silenced tumor suppressor genes, such as FHIT and WWOX[7]
7	GO:0007162_negative regulation of cell adhesion	CDKN2A , CDH13	hsa-let-7a, hsa-miR-200b/c	CDKN2A , CDH13, CDH1	
8	GO:0060191_regulation of lipase activity		hsa-miR-34b, hsa-miR-200c, hsa-miR-205	CALCA	
9	GO:0006275_regulation of DNA replication		hsa-miR-125a-5p, hsa-let-7a, hsa-miR-200b/c, hsa-miR-29b/c		
10	GO:0061061_muscle structure development	CHRNA1, ERBB2,	hsa-miR-125a-3p, hsa-miR-34b,	ERBB2, TWIST1	

		TWIST1	hsa-miR-155,<br>hsa-miR-200b/c,<br>hsa-miR-205,<br>hsa-miR-29a/b/c		
11	GO:0022008_neurogenesis	ASH1<br>YAP1,<br>VEGFC,<br>CHRNA1,<br>ERBB2,<br>KRAS,<br>DAPK3,<br>FAS,<br>NEUROD1/2,OPCML,<br>TWIST1,<br>CDH1,<br>DNMT3B,<br>NAB2,<br>SLIT2	hsa-miR-125a-5p,<br>hsa-miR-34b,<br>hsa-miR-155,<br>hsa-let-7a,<br>hsa-miR-200b/c,<br>hsa-miR-205,<br>hsa-miR-29a/b/c	ERBB2,<br>KRAS,<br>DAPK3,<br>FAS,<br>OPCML,<br>TWIST1,<br>CDH1,<br>DNMT3B,<br>SLIT2[13, 14]	miR-34b methylation up regulates c-Met[10]; Methylation makes miR-200c silence and down regulates TCF8 expression, then results in E-cadherin low expression, promote tumor deteriorate[5]
13	GO:0008543_fibroblast growth factor receptor signaling pathway		hsa-miR-200b/c,<br>hsa-miR-29a/b/c		
13	GO:0035107_appendage morphogenesis	HOXA9,<br>TWIST1	hsa-miR-125a-5p,<br>hsa-miR-34b,<br>hsa-miR-155,<br>hsa-miR-200b/c,<br>hsa-miR-205,<br>hsa-miR-29a/b/c	HOXA9,<br>TWIST1	
13	GO:0035108_limb morphogenesis	COX-2,<br>VEGFC,<br>HOXA9,<br>TWIST1	hsa-miR-125a-5p,<br>hsa-miR-34b,<br>hsa-miR-155,<br>hsa-miR-200b/c,<br>hsa-miR-205,<br>hsa-miR-29a/b/c	HOXA9,<br>TWIST1	
15	GO:0001568_blood vessel development	VEGFC,<br>IL8,<br>ERBB2,<br>CDH13,<br>CDX2,<br>SFRP1,<br>EGR1,<br>VEGFA,<br>SLIT2	hsa-miR-34b,<br>hsa-miR-155,<br>hsa-miR-200b/c,<br>hsa-miR-205,<br>hsa-miR-29a/b/c	IL8,<br>ERBB2,<br>CDH13,<br>CDX2,<br>SFRP1,<br>SLIT2	In lung cancer cells, EGR1 activates VEGFA expression[11]
16	GO:0005576_extracellular region	VEGFC,<br>IL8,<br>TNF,<br>FAS,<br>TIMP3 ,<br>LAMA3 ,<br>LAMB3 ,<br>LAMC2 ,<br>TMEFF2,<br>CDH13,<br>SFRP1,<br>EGFL7,<br>KLK13,<br>MMP1,<br>VEGFA,<br>SLIT2[8, 11, 15]	hsa-miR-125a-5p,<br>hsa-miR-125a-3p,<br>hsa-miR-34b,<br>hsa-miR-155,<br>hsa-let-7a,<br>hsa-miR-200b/c,<br>hsa-miR-205,<br>hsa-miR-29a/b/c	IL8,<br>TNF,<br>FAS,<br>TIMP3 ,<br>TMEFF2,<br>CDH13,<br>SFRP1,<br>CALCA,<br>MMP1,<br>SLI[15]	miR-200c was silented by methylation, down regulated TCF8 and E-cadherin, involving in lung cancer deteriorate[6]. KLK13 was up regulated by demethylation in its promoter, involving in tumor invasion;<br>SLIT2 low expression can down regulate beta-catenin and E-cadherin[16].
17	GO:0050793_regulation of developmental process	VEGFC,<br>NFKB1,<br>NFKBIA,<br>NFKBID,	hsa-miR-125a-5p,<br>hsa-miR-125a-3p,<br>hsa-miR-34b,<br>hsa-miR-155,	NFKB1,<br>TNF,<br>ERBB2,<br>CDKN2A ,	Up regulation of miR-29make sure normal patterns of DNA methylation, induces reexpression of methylation -silenced tumor suppressor

		TNF,<br>KEAP1,<br>CHRNA1,<br>ERBB2,<br>CDKN2A ,<br>FAS,<br>LAMA3 ,<br>NEUROD1,<br>CDX2,<br>SFRP1,<br>TWIST1,<br>DNMT3B,<br>VEGFA,<br>SLIT2[11, 17]	hsa-miR-200b/c,<br>hsa-miR-205,<br>hsa-miR-29 a/b/c[7, 12]	FAS,<br>LAMA3 ,<br>SFRP1,<br>TWIST1,<br>CALCA,<br>CDH1,<br>DNMT3B,<br>SLIT2[14]	genes, such as FHIT and WWOX[7]
18.5	GO:0010648_negative regulation of cell communication	NFKBIA,<br>NFKBID,<br>SFRP1,<br>WWOX,<br>SLIT2	hsa-miR-125a-3p,<br>hsa-miR-155,<br>hsa-let-7a,<br>hsa-miR-200b/c,<br>hsa-miR-205,<br>hsa-miR-29a/b/c	SFRP1,<br>SLIT2	Up regulation of miR-29 make sure normal patterns of DNA methylation, induces reexpression of methylation silenced tumor suppressor genes, such as FHIT and WWOX[7]
18.5	GO:0023057_negative regulation of signaling	NFKBIA,<br>NFKBID,<br>SFRP1,<br>WWOX,<br>SLIT2	hsa-miR-125a-3p,<br>hsa-miR-155,<br>hsa-let-7a,<br>hsa-miR-200b/c,<br>hsa-miR-205,<br>hsa-miR-29a/b/c	SFRP1,<br>SLIT2	
20	GO:0019216_regulation of lipid metabolic process	NFKB1,<br>TNF		NFKB1,<br>TNF	

Reference

1. Babbar N, H.A., Huang Y, Casero RA Jr., *Tumor necrosis factor alpha induces spermidine/spermine N1-acetyltransferase through nuclear factor kappaB in non-small cell lung cancer cells.* J Biol Chem., 2006. **281(34)**: p. 24182-92.
2. Nakanaga T, N.J., Ueki IF, Koff JL, Shao MX, *Regulation of interleukin-8 via an airway epithelial signaling cascade.* Am J Physiol Lung Cell Mol Physiol, 2007. **292(5)**: p. 1289-96.
3. Greenman, C., Stephens, P., Smith, R., Dalgliesh, G. L., Hunter, C., Bignell, G.,Davies, H., Teague, J., Butler, A., Stevens, C. and et al., *Patterns of somatic mutation in human cancer genomes.* Nature, 2007. **446**: p. 153-158.
4. Lili JIANG, Q.Z., Jihong CHANG, Xueshan QIU, Enhua WANG, *hsa-miR-125a-5p Enhances Invasion Ability in Non-Small Lung Carcinoma Cell Lines.* Chin J Lung Cancer, 2009. **12(9)**: p. 951-955.
5. Ceppi P, M.G., Kumarswamy R, Rapa I, Scagliotti GV, Papotti M, Allgayer H., *Loss of miR-200c expression induces an aggressive, invasive, and chemoresistant phenotype in non-small cell lung cancer.* Mol Cancer Res., 2010. **8(9)**: p. 1207-16.
6. Hurteau GJ, C.J., Spivack SD, Brock GJ., *Overexpression of the microRNA hsa-miR-200c leads to reduced expression of transcription factor 8 and increased expression of E-cadherin.* Cancer Res., 2007. **67(17)**: p. 7972-6.
7. Fabbri M, G.R., Cimmino A, Liu Z, Zanesi N, Callegari E, Liu S, Alder H, Costinean S, Fernandez-Cymering C, Volinia S, Guler G, Morrison CD, Chan KK, Marcucci G, Calin GA, Huebner K, Croce CM., *MicroRNA-29 family*

reverts aberrant methylation in lung cancer by targeting DNA methyltransferases 3A and 3B. Proc Natl Acad Sci U S A., 2007. **104(40)**: p. 15805-10.

8. Ding, L., Getz, G., Wheeler, D. A., Mardis, E. R., McLellan, M. D., Cibulskis, K.,Sougnez, C., Greulich, H., Muzny, D. M., Morgan, M. B. and et al., *Somatic mutations affect key pathways in lung adenocarcinoma.* Nature, 2008. **455(7216)**: p. 1069-1075.
9. X Xiang, X.Z., S Ju, S Zhang, H Jiang, J Mu, L Zhang, D Miller, W Grizzle and H.-G. Zhang, *miR-155 promotes macroscopic tumor formation yet inhibits tumor dissemination from mammary fat pads to the lung by preventing EMT.* Oncogene, 2011. **30(31)**: p. 3440-53.
10. Watanabe K, E.N., Hamano E, Sunohara M, Kawakami M, Kage H, Kitano K, Nakajima J, Goto A, Fukayama M, Nagase T, Yatomi Y, Ohishi N, Takai D., *Genome structure-based screening identified epigenetically silenced microRNA associated with invasiveness in non-small-cell lung cancer.* Int J Cancer., 2011. **epub ahead of print**.
11. Jorgen WESCHE1, K.H. and E.M. HAUGSTEN, *Fibroblast growth factors and their receptors in cancer.* Biochem J., 2011. **437**: p. 199-213.
12. Nozomu Yanaihara, N.C., Elise Bowman, Masahiro Seike, Kensuke Kumamoto, Ming Yi, Robert M. Stephens, Aikou Okamoto, Jun Yokota, Tadao Tanaka, George Adrian Calin, Chang-Gong Liu, Carlo M. Croce and C.C. Harris, *Unique microRNA molecular profiles in lung cancer diagnosis and prognosis.* Cancer Cell, 2006. **9(3)**: p. 189-198.
13. Belinsky, S.A., *Gene-promoter hypermethylation as a biomarker in lung cancer.* Nature Reviews Cancer, 2004. **4**: p. 707-717.
14. Selamat SA, G.J., Joshi AD, Fyfe MN, Campan M, Siegmund KD, Kerr KM, Laird-Offringa IA., *DNA methylation changes in atypical adenomatous hyperplasia, adenocarcinoma in situ, and lung adenocarcinoma.* PLoS One., 2011. **6(6)**: p. e21443.
15. Tellez CS, J.D., Do K, Bernauer AM, Thomas CL, Damiani LA, Tessema M, Leng S, Belinsky SA., *EMT and stem cell-like properties associated with miR-205 and miR-200 epigenetic silencing are early manifestations during carcinogen-induced transformation of human lung epithelial cells.* Cancer Res., 2011. **71(8)**: p. 3087-97.
16. Chou RH, L.S., Wen HC, Wu CW, Chang WS, *Epigenetic activation of human kallikrein 13 enhances malignancy of lung adenocarcinoma by promoting N-cadherin expression and laminin degradation.* Biochem Biophys Res Commun, 2011. **409(3)**: p. 442-7.
17. Toyooka S, M.T., Soh J, Aokage K, Yamane M, Oto T, Kiura K, Miyoshi S., *Molecular oncology of lung cancer.* Gen Thorac Cardiovasc Surg, 2011. **59(8)**: p. 527-37.
18. Weiss, J., Sos, M. L., Seidel, D., Peifer, M., Zander, T., Heuckmann, J. M., Ullrich, R. T., Menon, R., Maier, S., Soltermann, A. and et al., *Frequent and focal FGFR1 amplification associates with therapeutically tractable FGFR1*

dependency in squamous cell lung cancer. Sci. Transl. Med., 2010. **2**: p. 62-93.

19. Warburton, D., El-Hashash, A., Carraro, G., Tiozzo, C., Sala, F., Rogers, O., De Langhe, S., Kemp, P. J., Riccardi, D., Torday, J. and et al., *Lung organogenesis.* Curr. Top. Dev. Biol., 2010. **90**: p. 73-158.
20. M., F., *The search for improved systemic therapy of non-small cell lung cancer--what are today's options?* Lung Cancer, 2011. **72(3)**: p. 265-70.
21. Lili JIANG, Q.Z., Hongji CHANG, Xueshan QIU, Enhua WANG, *hsa-miR-125a-5p Enhances Invasion in Non-small Cell Lung Carcinoma Cell Lines by Upregulating Rock-1.* Chin J Lung Cancer, 2009. **12(10)**: p. 1069-1073.
22. Helen Davies, C.H., Raffaella Smith, *Somatic Mutations of the Protein Kinase Gene Family in Human Lung Cancer.* Cancer Res., 2005. **65(17)**: p. 7591-7595.
23. Liu L, W.J., Wu C, Wang Y, Zhong R, Zhang X, Tan W, Nie S, Miao X, Lin D, *A functional polymorphism (-1607 1G→2G) in the matrix metalloproteinase-1 promoter is associated with development and progression of lung cancer.* Cancer, 2011. **epub ahead of print**: p. e26154.
